# Human‐Centric, Three Dimensional Micro Light‐Emitting Diodes for Cosmetic and Medical Phototherapy

**DOI:** 10.1002/advs.202416716

**Published:** 2025-02-17

**Authors:** Ki Yun Nam, Min Seo Kim, Jaehun An, Seongwook Min, Jae Hee Lee, Jae Sung Park, Chang‐Hun Huh, Seok Hyun Yun, Keon Jae Lee

**Affiliations:** ^1^ Department of Materials Science and Engineering Korea Advanced Institute of Science and Technology (KAIST) 291 Daehak‐ro, Yuseong‐gu Daejeon 34 141 Republic of Korea; ^2^ School of Electrical Engineering Graduate School of Semiconductor Technology Korea Advanced Institute of Science and Technology (KAIST) 291 Daehak‐ro, Yuseong‐gu Daejeon 34 141 Republic of Korea; ^3^ Querrey‐Simpson Institute for Bioelectronics Northwestern University Evanston IL 60 208 USA; ^4^ Yonsei Myview Clinic 301, Sadang‐ro, Dongjak‐gu Seoul 0 7008 Republic of Korea; ^5^ Department of Dermatology Seoul National University Bundang Hospital (SNUBH) 173–82, Gumi‐ro, Bundang‐gu Seongnam 13 620 Republic of Korea; ^6^ Harvard Medical School and Wellman Center for Photomedicine Massachusetts General Hospital Boston MA 0 2114 USA

**Keywords:** micro light‐emitting diodes (µLEDs), phototherapy, shape morphing, self‐adaptive, multilayered spatiotemporal mapping

## Abstract

Phototherapy based on micro light‐emitting diodes (µLEDs) has gained enormous attention in the medical field as a patient‐friendly therapeutic method due to its advantages of minimal invasiveness, fewer side effects, and versatile device form factors with high stability in biological environment. Effective cosmetic and medical phototherapy depends on deep light penetration, precise irradiation, and simultaneous multi‐site stimulation, facilitated by three‐dimensional (3D) optoelectronics specifically designed for complex human matters, defined here as 3D µLEDs. This perspective article aims to present the functionalities and strategies of 3D µLEDs for human‐centric phototherapy. This study investigates the effectiveness of phototherapy enabled by three key functionalities such as shape morphing, self‐adaptation, and multilayered spatiotemporal mapping of 3D µLEDs. Finally, this article provides future insights of 3D µLEDs for human‐centric phototherapy applications.

## Graphical abstract

This work presents the three‐dimensional (3D) micro light‐emitting diodes (3D µLEDs) for human‐centric cosmetic and medical phototherapy. The functionalities of 3D µLEDs such as shape morphing, self‐adaptation, and multilayered spatiotemporal mapping maximize the phototherapeutic effect compared to conventional two‐dimensional (2D) photonic devices. By providing perspectives on human phototherapy applications, 3D µLEDs pave the way for future human‐centric phototherapy.

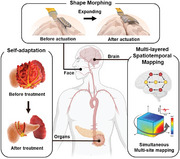

## 1 Introduction

As the global population ages, the desire to maintain a youthful, beautiful appearance and a healthy life has become increasingly prominent. However, the accelerating progression of aging problems, including skin aging and hair loss, along with diseases such as neurological disorders and cancer, poses significant obstacles to achieving this aspiration.^[^ ^1^, ^2^, ^3^, ^4^, ^5^, ^6^, ^7^ ^]^ The medications used for cosmetic therapy face limitations in precisely targeting affected areas, resulting in side effects such as scalp and skin irritation, depression, and sexual dysfunction.^[^ ^8^, ^9^, ^10^, ^11^ ^]^ Moreover, conventional disease treatment methods such as invasive surgery, radiation therapy, and chemotherapy may lead to adverse effects including immune system impairment and second malignancies due to late toxicity.^[^ ^12^, ^13^, ^14^, ^15^, ^16^, ^17^, ^18^, ^19^, ^20^ ^]^ To overcome these drawbacks, inorganic micro light‐emitting diode (µLED) based phototherapy has been spotlighted as a patient‐friendly therapeutic approach, offering advantages such as minimal invasiveness, fewer side effects, versatile device form factor, and excellent device stability in the biological environment.^[^ ^21^, ^22^, ^23^, ^24^, ^25^, ^26^ ^]^

**Figure 1:**
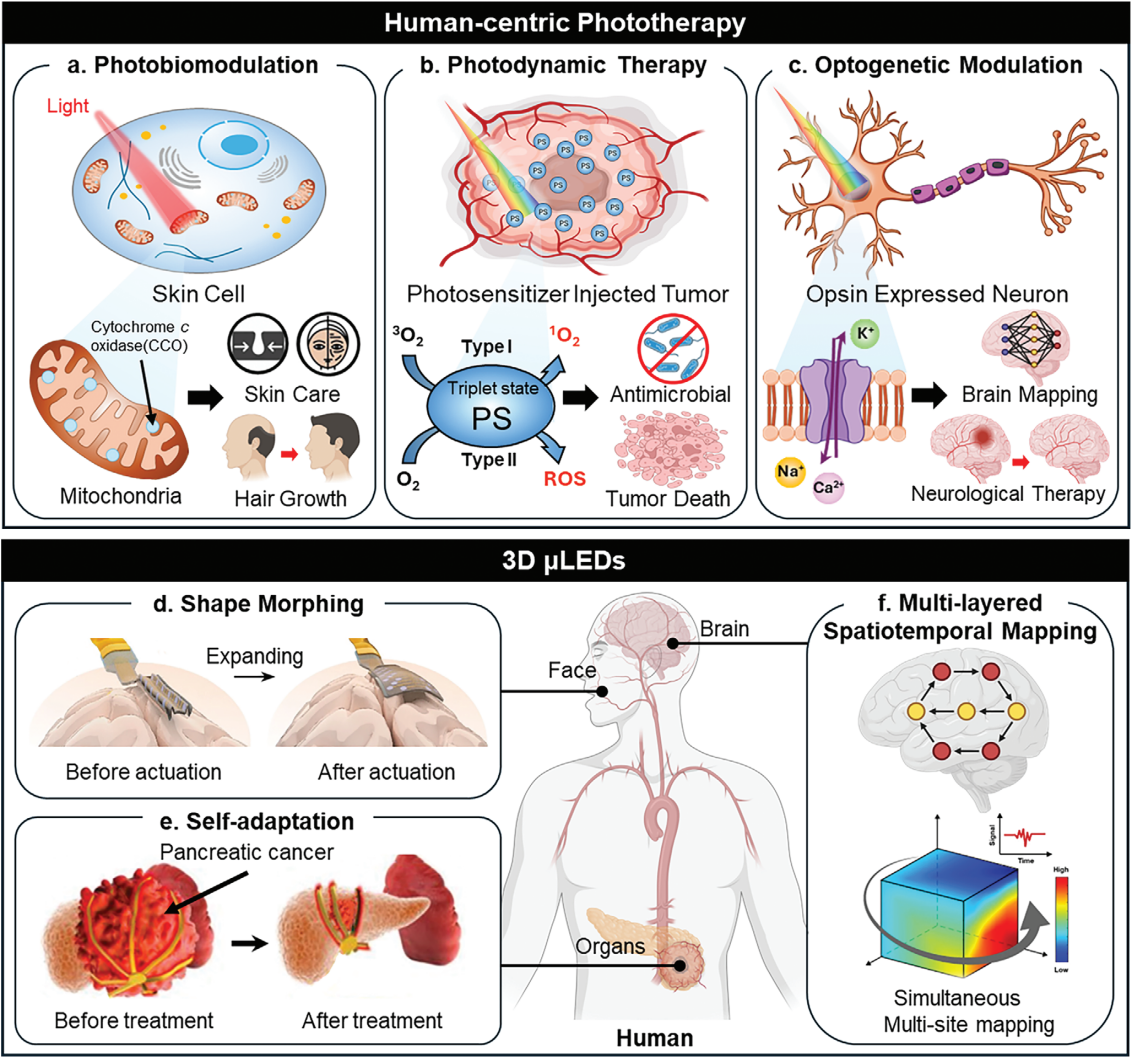
Schematic illustration of the mechanism of human‐centric phototherapy including photobiomodulation (PBM) therapy, photodynamic therapy (PDT), and optogenetic modulation with three functionalities and strategies of three‐dimensional (3D) micro light‐emitting diodes (µLEDs). a) The mechanism of PBM therapy, b) PDT, and c) optogenetic modulation. d) Shape morphing functionality of 3D µLEDs for human‐centric phototherapy. Reproduced with permission.^[^ ^53^ ^]^ Copyright 2024, Springer Nature. e) Self‐adaptation functionality of 3D µLEDs for human‐centric phototherapy. Reproduced with permission.^[^ ^52^ ^]^ Copyright 2024, Wiley‐VCH. f) Multilayered spatiotemporal mapping functionality of 3D µLEDs for human‐centric phototherapy. Reproduced with permission.^[^ ^54^ ^]^ Copyright 2022, Springer Nature. Some parts of Figure 1 were created with BioRender.com.

Phototherapy includes photobiomodulation (PBM) therapy, photodynamic therapy (PDT), and optogenetic modulation, enabling cosmetic and disease treatments through biological and photochemical reactions when light interacts with organs and tissues. PBM therapy, also referred to as low‐level light therapy, reduces inflammation, promotes healing, and restores cellular biological functions using red and near‐infrared light.^[^ ^27^, ^28^, ^29^, ^30^ ^]^ **Figure** **1**a shows the mechanism and therapeutic purposes of PBM therapy, which is driven by the optical stimulation of mitochondria to promote skin care and hair growth.^[^ ^22^, ^31^, ^32^, ^33^ ^]^ Although several researches on PBM therapy utilizing µLEDs have been demonstrated, two‐dimensional (2D) optical devices exhibit limited therapeutic effects due to shallow light penetration.^[^ ^34^, ^35^ ^]^ PDT is a medical treatment that induces a photochemical reaction through the interaction between a photosensitizer (PS) and light, selectively destroying target cells. Figure 1b illustrates the mechanism and treatment outcomes of PDT, caused by the PS excitation, leading to antimicrobial and tumor death.^[^ ^36^, ^37^, ^38^, ^39^, ^40^, ^41^, ^42^ ^]^ Despite several efforts regarding µLED‐based PDT, the treatment efficacy of 2D photodevice remains restricted due to inaccurate light irradiation.^[^ ^43^, ^44^ ^]^ Optogenetic modulation is a therapeutic approach that controls the activity of genetically modified cells through opsins. Figure 1c presents the optogenetic modulation process with its treatment objectives, where light is applied to opsin‐expressed neurons, enabling the treatment of neurological disorders and the investigation of brain circuits.^[^ ^22^, ^45^, ^46^, ^47^ ^]^ Although several studies related to optogenetic modulation based on µLEDs were proved, existing 2D optoelectronics are incapable of concurrent optical stimulation of surface and interior brain regions, presenting limitations for exploring complex brain circuits and treating neurological disorders across a wide area.^[^ ^48^, ^49^, ^50^, ^51^ ^]^

To overcome these structural limitations, three‐dimensional (3D) µLEDs, defined as µLED‐based optoelectronics with diverse 3D architectures have emerged, providing deep light penetration depth, precise light irradiation, and simultaneous photostimulation of both surface and deep brain regions.^[^ ^33^, ^52^ ^]^ These 3D µLEDs offer three key functionalities: i) shape morphing,^[^ ^53^ ^]^ ii) self‐adaptation,^[^ ^52^ ^]^ and iii) multilayered spatiotemporal mapping^[^ ^54^ ^]^ to achieve effective phototherapy. Figure 1d displays the schematic of the shape morphing capability of 3D µLEDs, enabling conformal contact with static complex 3D curvatures after actuation for efficient light delivery to tissues and organs without significant light loss. For example, efficacious PBM therapy can be achieved through deep light penetration on static facial skin by utilizing this. Figure 1e illustrates the functionality of 3D µLEDs that enable real‐time self‐adaptation to dynamic shape deformations of organs and tissues for sustained high‐efficiency light irradiation. With this capability, 3D µLEDs can respond to reversible shape changing, enabling effective PDT by irradiating accurate lights to target tumors. As shown in Figure 1f, multisite mapping can be achieved through multilayered spatiotemporal mapping functionality of 3D µLEDs regardless of anatomical depth. Integrating 3D µLEDs with diverse functionalities into human organs and tissues promises to advance human‐centric phototherapy.

This review aims to present the recent progress in 3D µLEDs for highly effective human‐centric phototherapy in terms of functionality and strategy of 3D µLEDs (Figure 1). In detail, Section (2) mainly describes therapeutic effects in cosmetic and medical phototherapy utilizing µLED‐based soft optoelectronics, as well as transfer technologies for their fabrication. Section (3) explains the functionalities of 3D µLEDs with diverse strategies for enhancing these capabilities. Finally, we discuss the practical challenges of applying 3D µLEDs to humans and present a new perspective to address these issues.

## 2 Phototherapy Applications via µLEDs

Phototherapy enables disease treatment, aging problem improvement, and nervous system control through interactions including photochemical reactions and biological responses between light and tissues. Effective phototherapy requires selecting a light source that aligns with the treatment purpose and possesses appropriate optical characteristics including wavelength and FWHM.^[^ ^32^, ^55^, ^56^ ^]^ Notably, the wavelength of light plays a vital role in determining penetration depth within tissues. When light is illuminated in biological tissues, photons are absorbed and scattered by cells and proteins, showing different light penetration depths based on the wavelength. Light with wavelengths of 400–500 nm penetrates only to the surface of tissues due to light absorption by molecules including hemoglobin and melanin within the tissue. On the other hand, light with wavelengths above 600 nm reaches deeper sites of tissues due to its low absorption rates.^[^ ^22^ ^]^ Moreover, light sources require stable operation without performance degradation in biological environments. Notably, light sources that directly contact soft tissues and organs require the assurance of safety without causing tissue damage. **Table** **1** shows the comparison of characteristics and adverse effects of three types of light sources used in phototherapy.^[^ ^57^, ^58^, ^59^, ^60^, ^61^ ^]^

**Table 1:** Comparison of the characteristics and adverse effects of light sources used in phototherapy.

		Lasers	IPL	LEDs		
				OLEDs	QLEDs	Inorganic µLEDs
Optical characteristics	Coherence	Coherent (monochromatic)	Noncoherent (polychromatic)	Noncoherent (polychromatic)		
	Output power	Proportional to current above the threshold	Proportional to current above the threshold	Linearly proportional to drive current		
	FWHM	0.0001–10 nm	≈100 nm	≈100 nm	20–50 nm	≈10 nm
	Power efficiency	Low	Low	Low	High	High
	Life span	High	High	Low	Low	High
In vivo performance	Safety	–	–	High	Low	High
	Long‐term stability	–	–	Low	Low	High
	Heat generation	–	–	Moderate	Low	Low
	Biocompatibility	–	–	Moderate	Low	High
Cost		High	High	Low	Low	High
Adverse effects (Applications)		Erythema, pruritus (photobiomodulation (PBM) therapy‐skin care), mild headache (PBM therapy‐hair loss treatment, optogenetic modulation)	Transient erythema and edema, mild tining, burning sensation, post‐inflammatory hyperpigmentation (PBM therapy‐skin care)	Low temperature burns (PBM therapy‐skin care)	‐	‐

Lasers emit monochromatic and coherent light with high collimation and a narrow wavelength range through optical amplification. These optical characteristics enable phototherapy requiring precise targeting. Despite these advantages, lasers inherently face challenges such as low power efficiency and risks including burns, infections, and scarring caused by excessive heat generation.^[^ ^56^, ^62^ ^]^

Intense pulsed light (IPL) uses flashlamps to emit polychromatic and incoherent high‐intensity pulsed light with tunable pulse width. The emission wavelength of IPL primarily lies within the range of 500 to 1200 nm and can be selectively filtered using cut‐off filters based on skin type and injuries.^[^ ^63^ ^]^ The working principle of IPL in phototherapy is based on the selective photothermalmolysis effects on biological tissues. Although successful research on phototherapy using IPL has been reported, critical issues regarding side effects such as scarring, burning sensation, and post‐inflammatory hyperpigmentation are still outstanding.^[^ ^61^, ^64^ ^]^

Light‐emitting diodes (LEDs) have emerged as a strong candidate for light sources in phototherapy, addressing the challenges of lasers and IPL. LEDs are semiconductor devices that emit incoherent light, offering high‐cost efficiency and compact size, making them suitable for compact home care phototherapeutic devices.^[^ ^62^ ^]^ Although diverse studies on phototherapy utilizing organic LEDs (OLEDs) and quantum‐dot LEDs (QLEDs) have been actively reported, they face limitations in safety and long‐term stability.^[^ ^59^, ^65^, ^66^, ^67^ ^]^ OLEDs encounter challenges such as chemical toxicity, degradation of organic materials during prolonged operation, and reduced device performance in high‐temperature and high‐humidity environments. Moreover, due to issues such as quantum dot toxicity and degradation of quantum dot efficiency during extended operation, QLEDs are constrained by limitations in safety and long‐term stability.

Due to these challenges, the inorganic‐based µLEDs have been spotlighted as the most suitable light source for efficacious phototherapy based on their excellent durability and low toxicity. Moreover, the µLEDs exhibit excellent optical properties such as high external quantum efficiency (EQE), narrow FWHM, and high power efficiency due to their high crystal quality of inorganic materials.^[^ ^26^, ^50^, ^68^ ^]^ These outstanding optical characteristics ensure their superior in vivo performance including high long‐term stability and high safety. Notably, the high operation efficiency of µLEDs prevents the conversion of supplied power into heat, maintaining low heat generation during operation in biological environment. Moreover, µLEDs provide high spatial resolutions and flexibility due to their tiny size and thickness, offering precise light irradiation and biocompatibility with soft tissues.^[^ ^25^, ^48^ ^]^

The outstanding optical properties of µLEDs stem from the emission layer known as multiple quantum wells (MQWs), which maximize emission efficiency by alternating semiconductor materials with different bandgaps to increase recombination probability. As shown in **Table** **2**, the combination of materials in MQWs determines the optical properties of µLEDs, making careful consideration essential. Although µLEDs exhibit remarkable emission efficiency, efficiency degradation due to dominant nonradiative recombination occurs at high‐level current injection.^[^ ^69^ ^]^ Moreover, Joule heating caused by nonradiative recombination leads to self‐heating‐induced bandgap shrinkage, which results in emission wavelength redshift and reduced phototherapy efficacy.^[^ ^70^, ^71^, ^72^ ^]^ Therefore, thermal management techniques of µLEDs such as structure optimization, flip‐chip packaging, and using heat sinks are crucial for safe and highly efficacious phototherapy, preventing wavelength shift and minimizing tissue thermal damage.

**Table 2:** Comparison of optical properties based on material combinations in multiple quantum wells (MQWs) of micro light‐emitting diodes (µLEDs).

Materials	Color	Wavelength [nm]	Size [µm^2^]	FWHM [nm]	Power density	EQE_max_ [%]	Refs.
AlGaN MQWs	DUV	275	20 × 20	11.4	86 W cm^−2^ @ 1300 A cm^−2^	1.2 @ 99.52 A cm^−2^	[171]
InGaN MQWs	UV	370	–	10	–	–	[172]
InGaN/GaN MQWs	Blue	446	50 × 50	25	3 W cm^−2^ @ 25 A cm^−2^	–	[68]
InGaN/GaN MQWs	Blue	447	100 × 100	–	–	48.6 @ 10 A cm^−2^	[173]
InGaN MQWs	Green	520	80 × 80	30	140.6 W cm^−2^ @ 800 A cm^−2^	23.8 @ 4.7 A cm^−2^	[174]
AlGaInP MQWs	Red	630	25 × 17	21	‐	–	[175]
AlGaInP MQWs	Red	638	50 × 50	11	2.5 W cm^−2^ @ 80 A cm^−2^	–	[48]
AlGaAs MQWs	Red	649	100 × 100	24	–	–	[176]
InGaAl/AlGaAs MQWs	NIR	864	–	25	0.07068 W @ 0.5 A	–	[177]

### 2.1 Transfer Technologies of µLEDs for Soft Optoelectronics

The fabrication of µLEDs typically requires high‐temperature epitaxial growth on rigid substrates, necessitating a subsequent transfer process to flexible and stretchable substrates for integration into soft optoelectronic systems.^[^ ^73^, ^74^ ^]^ However, this transfer process is constrained by a critical trade‐off between yield and precision, significantly limiting the scalability and reliability of µLED‐based applications, such as phototherapy. To overcome these challenges, various transfer technologies have been developed, including elastomeric, electrostatic, and electromagnetic transfer methods.^[^ ^75^, ^76^, ^77^ ^]^ Among these, elastomeric transfer, relying on the use of an elastomeric stamp and kinetically controlled van der Waals forces to transfer inorganic microstructures such as GaN, Si, and mica, has been extensively studied. By carefully controlling the peeling rate of the elastomeric stamp, precise and selective transfers can be achieved.^[^ ^75^ ^]^ Despite its promise, this technique faces persistent challenges such as limited stamp reusability, potential damage to µLED chips during transfer, low transfer selectivity for specific patterns, and misalignment issues during large‐scale assembly.

**Figure 2:**
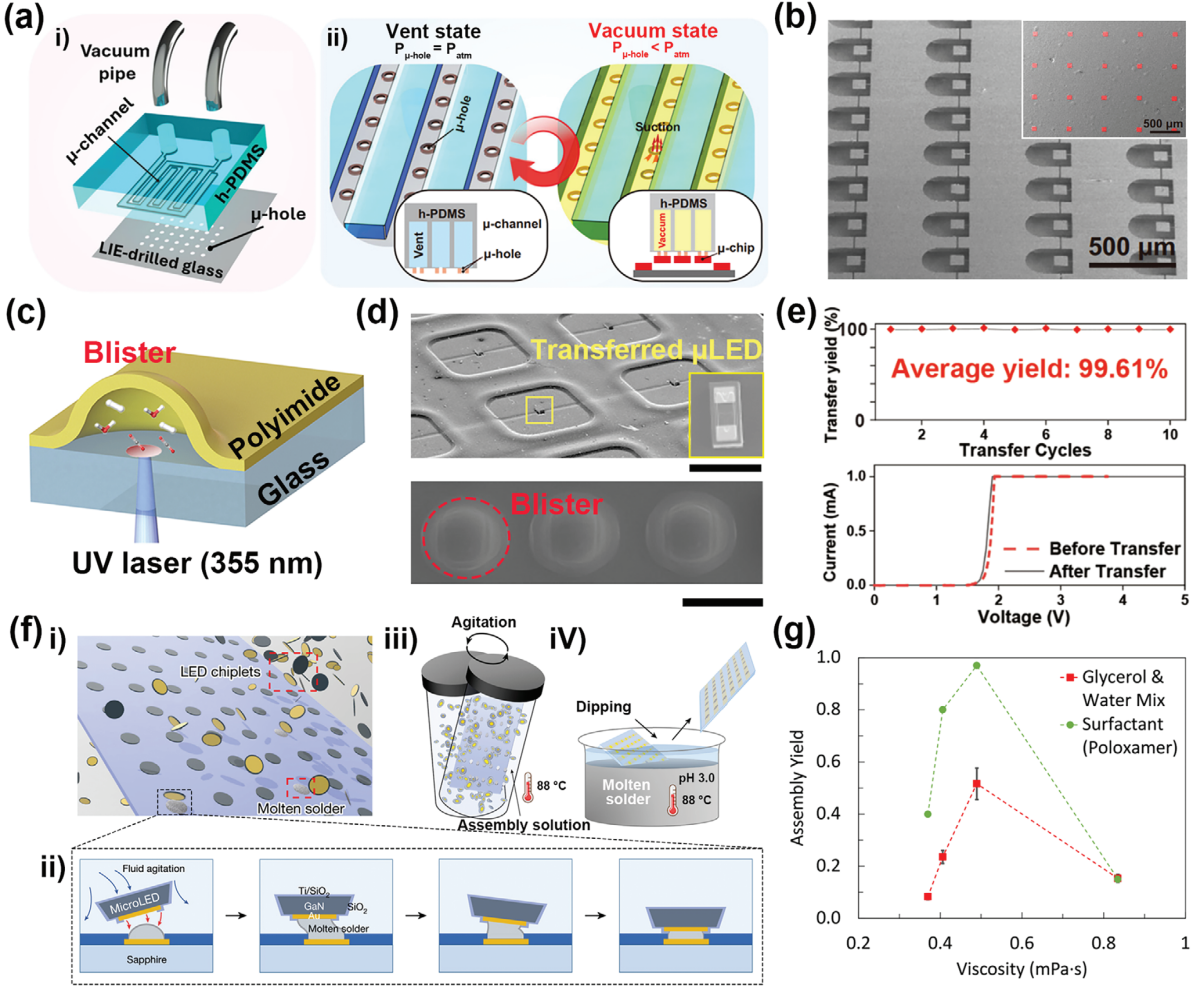
Transfer technologies of micro light‐emitting diodes (µLEDs) for soft optoelectronics. a) Concept of micro‐vacuum assisted selective transfer printing (µVAST). i) An exploded scheme of the vacuum controllable module (VCM) with interdigitated µ‐channels and µ‐holes arranged with regular distances, ii) Pressure change inside the µ‐channel during the µVAST process. b) SEM image of freestanding µLED arrays on GaAs substrate. Inset: Colored SEM image of transferred µLEDs on the final substrate. Reproduced with permission.^[^ ^26^ ^]^ Copyright 2023, Springer Nature. c) Schematic illustration of the blister‐assisted laser‐induced forward transfer (BA‐LIFT) mechanism. The blister is formed by gas formation and expansion through partial decomposition in the laser‐irradiated part of the polyimide (PI). d) SEM image of the µLEDs transferred to FPCB (upper), dynamic release layer (DRL) with blister formed by UV laser (lower). e) Average transfer yield during 10 transfer cycles (upper), *I*–*V* characteristics of µLEDs before and after BA‐LIFT (lower). Reproduced with permission.^[^ ^33^ ^]^ Copyright 2024, Wiley‐VCH. f) Schematic of the fabrication of µLED lighting panel by FSA. i) schematic showing a region near the substrate during the agitation process, ii) Enlarged view of a binding site showing the assembly process, iii) Schematic of agitation process, iv) Schematic of the dip‐soldering process whereby solder bumps are selectively formed on Au patterns on the substrate. g) Self‐assembly yield versus viscosity characteristics. Reproduced with permission.^[^ ^81^ ^]^ Copyright 2023, Springer Nature.

To address these limitations, numerous transfer technologies such as micro‐vacuum assisted selective transfer (µVAST), laser‐induced forward transfer (LIFT), and fluidic self‐assembly (FSA) have emerged. **Figure** **2**a presents the composition of the vacuum controllable module (VCM) and the principal transfer mechanism utilizing VCM. The VCM is composed of hard PDMS with negatively patterned µ‐channels, a glass substrate with a µ‐hole array formed by the laser‐induced etching (LIE) process, and a pipe (Figure 2a‐i). The main transfer mechanism is the individual control of vacuum levels inside the µ‐channels, enabling the selective and massive transfer of µLEDs (Figure 2a‐ii). Figure 2b exhibits the freestanding µLED arrays suspended by micro‐bridges (µ‐bridges) to apply vacuum suction force. During the pick‐up process, vacuum suction force detaches µLEDs from the donor wafer as µ‐bridges fracture.^[^ ^26^ ^]^

The LIFT method utilizes an interaction between a laser beam and a dynamic release layer (DRL) that temporarily holds µLEDs.^[^ ^78^, ^79^, ^80^ ^]^ When the laser beam is applied to the interface between DRL and donor substrate, various reactions such as blister formation, ablation, and adhesion change occur that enable precise and selective transfer. Figure 2c shows the phenomenon of blister formation during the blister‐assisted LIFT (BA‐LIFT) process using UV laser (355 nm) and polyimide (PI) as DRL. The blister occurs when the laser beam is absorbed at the PI/glass interface, generating heat exceeding 1000 °C, which induces a partial decomposition, resulting in the formation of gases trapped at the interface. Figure 2d displays transferred µLEDs on the accurate location of the flexible substrate through BA‐LIFT. The blister formation leads the contact area of the µLEDs attached to the DRL to decrease, enabling the downward transfer of the chips to the target substrate. As shown in Figure 2e, BA‐LIFT achieved a high transfer yield of approximately 99.61% by optimizing the distance between the donor and receiver substrates. Moreover, there are almost no differences in the forward voltage of µLEDs before and after the transfer, indicating that the BA‐LIFT method offers a damage‐free process.^[^ ^33^ ^]^ These transfer printing methods provide fast, damage‐free, and high‐yield transfer of µLEDs, enabling the realization of form‐factor free optoelectronics for phototherapy.

To further advance µLED‐based phototherapy devices, transfer methods are required that are not only accurate and damage‐free but also capable of controlling numerous µLEDs to cover large areas of the human body. The FSA method employs fluidic mediums to align µLEDs on the binding sites of the final substrate without damage.^[^ ^81^, ^82^, ^83^ ^]^ Figure 2f exhibits the high yield, high throughput, and large‐scale FSA method, which facilitates momentum transfer from liquid to microchips during agitation by increasing the viscosity of the solution. The dip‐soldering process is carried out to form a binding site between the patterned Au on the sapphire substrate and the chiplet (Figure 2f‐i). After immersing the substrate and µLEDs in the solution, they are agitated manually to induce contact between solder and Au layers of µLEDs (Figure 2fii–iv). By increasing the viscosity of the solution, the momentum transfer from the liquid to the chiplets is enhanced, allowing the high‐yield assembly of µLEDs under 100 µm. Figure 2g shows the addition of 2 wt% of poloxamer, a high‐molecular‐weight polymer, improves viscosity and enables transferring more than 19 000 µLEDs with a yield of 99.88% in just 60 s.^[^ ^81^ ^]^ Although flexible optoelectronics utilizing the FSA method have not been demonstrated, the potential for their future development remains significant. With the advancement of these transfer technologies, the possibility of the utilization of µLEDs in cosmetic and medical phototherapy is significantly increasing.

### 2.2 Cosmetic Phototherapy

Appearance plays a vital role in communicating one's identity to others and reflects key factors influencing the perception of overall health. However, the world is facing global population aging, with an estimated 16% of the global population projected to be aged 65 by 2050, leading to an increase in individuals experiencing aging‐related issues such as skin aging and hair loss.^[^ ^84^ ^]^ Unlike the invisible signs of aging, hair loss and skin aging become more apparent over time, leading to a growing demand for convenient cosmetic therapies with minimal side effects.

Facial skin beauty significantly influences emotional and psychological health, ultimately affecting overall quality of life.^[^ ^85^ ^]^ As a result, interest in appearance has surged, and a survey reported that the proportion of women aged 18 to 24 who consider skin anti‐aging care important increased significantly from less than 20% in 2012 to over 50% in 2018.^[^ ^86^ ^]^ Skin aging such as deep wrinkles, pigmentation, and roughness is associated with the aging of skin cells and a decrease in collagen synthesis or an increase in collagen degradation.^[^ ^87^ ^]^ The cause of skin aging can be categorized into endogenous (or intrinsic) and exogenous (or extrinsic) factors. The modification of endogenous factors such as hormone processes, genetics, and cellular metabolism and the exposure of exogenous factors including UV radiation and pollution lead to cumulative structural and physiological alterations and progressive changes in each skin layer.

As shown in **Table** **3**, various therapeutic approaches including physical and chemical methods have emerged to achieve skin anti‐aging. Current physical and chemical therapies, such as ultrasound and drug injection, focus on localized tissue points to induce molecular vibrations that generate collagen denatures or employ invasive procedures to inhibit skin aging. However, these treatments have risks of thermal damage and infection during the treatment procedure, and the necessity for expensive specialized equipment and professional supervision limits their feasibility for cost‐effective home treatments.^[^ ^88^, ^89^ ^]^ Optical therapy using LEDs is attracting attention as a promising skin anti‐aging treatment because of its noninvasive and safe characteristics. However, the efficacy of LED‐based phototherapy remains controversial due to its low effectiveness.

**Table 3:** Comparison of the characteristics between three types of photobiomodulation (PBM) therapy methods.

Method	Subtype	Treatment efficacy	Risk	Homecare capability	Cost
Physical	Ultrasound	Good	Low‐high	Low‐moderate	Moderate‐high
	Microdermabrasion	Moderate	High	Low	Moderate
Chemical	Chemical peeling	Good	High	Low	Moderate‐high
	Drug injection	Good	Moderate‐high	Low	High
Optical	Low‐level laser therapy (LLLT)	Good	Moderate	Low	High
	LED therapy	Poor	Moderate	High	Low
	FSLED mask	Good	Low	High	Low

Hair loss, medically referred to as alopecia, can cause significant emotional distress including loss of self‐esteem, anxiety, and depression.^[^ ^90^, ^91^, ^92^ ^]^ The alopecia affects common people worldwide regardless of age or ethnicity. It is driven by various factors such as genetic predisposition, autoimmune factors, and micronutrient deficiencies.

Hair follicles have a three‐phase life cycle: anagen phase (hair growth), catagen phase (involuting), and telogen phase (resting). The anagen phase lasts 2 to 8 years and determines hair length as matrix cells at the base of the follicle continuously proliferate and differentiate to promote hair growth. The catagen phase is the transition stage between anagen and telogen which lasts for 3 to 4 weeks. The telogen phase is the final phase of the hair life cycle which lasts for 2 to 3 months, leading the follicles to lie dormant in a resting phase.^[^ ^93^, ^94^, ^95^ ^]^ In a typical scalp, about 90% of the hair follicles are in the anagen phase, while the remainder are in the telogen phase, with approximately 100 hairs shedding daily.

When the natural life cycle of hair follicles is disrupted, resulting in a shortened growth phase, it can be defined as hair loss. Among various types of hair loss, androgenetic alopecia (AGA), also known as male pattern hair loss (MPHL) or female pattern hair loss (FPHL), is the most common type of alopecia worldwide.^[^ ^96^, ^97^ ^]^ AGA is a polygenic condition characterized by differences in the age of onset, severity, and areas of hair loss, with men commonly experiencing hair thinning mainly in the temporal and vertex regions.^[^ ^98^ ^]^ AGA is primarily caused by the effects of androgen hormones including dihydrotestosterone (DHT) and testosterone on hair follicles, which induce hair follicle miniaturization and progression out of the anagen phase, leading to gradual hair thinning and hair loss.^[^ ^95^ ^]^ **Table** **4** shows various therapies that have emerged to treat AGA, including oral medications, topical treatments, and light‐based therapies. By inhibiting the conversion of testosterone to DHT via 5 α‐reductase enzyme, oral medications have shown excellent effects on AGA treatment. However, concerns about adverse effects are significant due to the difficulty in identifying precise targets.^[^ ^10^, ^99^, ^100^, ^101^, ^102^ ^]^

**Table 4:** Summary of the treatment mechanisms and characteristics of androgenetic alopecia (AGA) treatment methods.

Type of treatment	Source	Wavelength [nm]	Power	Treatment frequency	Total period [weeks]	Mechanism of treatments	Pros.	Cons.	Refs.
Oral medications	Finasteride	‐	‐	1 mg day^−1^	24	Inhibits the conversion of testosterone to DHT via 5‐α reductase enzyme (mainly Type 2)	Excellent therapeutic efficacy, convenient treatment	Requires consistent, long‐term treatment, high risk of side effects	[10]
	Dutasteride	‐	‐	0.5 mg day^−1^	24–48	Inhibits the conversion of testosterone to DHT via 5‐α reductase enzyme (Type 1, 2, and 3)			[11, 99, 100]
	Minoxidil	‐	‐	1–5 mg day^−1^	24	Unclear mechanism (acting as a vasodilator or Wnt/β‐catenin signaling pathway inducer)			[178, 179, 180]
	Spironolactone	‐	‐	100–200 mg day^−1^	24−48	Reduces total testosterone levels and blocks the androgen receptor (AR), preventing DHT produced from binding to the receptor	Convenient treatment, fewer sexual side effects		[181, 182]
Topical medications	Minoxidil	‐	‐	5% MS, 2 mL day^−1^ with half a capful of 5% MF 2% MS, 2 mL day^−1^ with half a capful of 5% MF	24–48	Increased blood flow to the scalp through vasodilation, promotes cell growth through activation of potassium channels minoxidil sulphate (metabolite) promotes hair follicle cell proliferation and survival	High therapeutic efficacy.convenience treatment, fewer side effects than oral medications	Requires consistent, long‐term treatment, high risk of side effects	[102, 183, 184]
	Finasteride	‐	‐	0.25% Finasteride, 100–200 µL day^−1^	24–48	Inhibits the conversion of testosterone to DHT via 5‐α reductase enzyme			[8, 101]
Optical treatments	Laser	650	5 mW	20 min day^−1^ for other days	38	Activation of Wnt/b‐catenin signaling pathway through ROS and ATP, which were generated through the interaction between light and CCOs in mitochondria	Higy therapeutic efficacy Fewer side effects than Oral and Topical medications	Variations in light parameters can lead to differences in treatment results	[185]
	Laser	650	5 mW	30 min day^−1^ for other days	24				[183]
	Laser	1540	6 mJ	10 time (2 weeks interval)	20				[186]
	Laser	1550	6 mJ	10 time (2 weeks interval)	20				[187]
	LED	633 ± 10	80 mW cm^−^^2^	10 min day^−1^, 3 day week^−1^	24				[188]
	µLED	650	5 mW mm^−^^2^	15 min day^−1^, everyday	3				[34]

Recently, a novel approach utilizing tannic acid, a polyphenol, as an adhesion mediator has been reported, demonstrating its efficacy in delivering functional molecules such as salicylic acid, niacinamide, and dexpanthenol to hair follicles, enhancing hair health and mitigating hair loss.^[^ ^103^ ^]^ Furthermore, an AGA treatment method using self‐assembling micelle inhibitory RNA (SAMiRNA) nanoparticle‐type siRNA has been developed to reduce androgen receptor (AR) levels, resulting in the inhibition of DHT binding.^[^ ^104^ ^]^ In clinical trials, delivering SAMiRNA to human follicle dermal papilla cells and hair follicles demonstrated an effective reduction of AR mRNA and protein levels. Although clinical trials using these two methods have demonstrated their high therapeutic effects for hair loss treatment, the risk of potential side effects still remains.

#### 2.2.1 PBM Therapy for Skin Aging

**Figure 3:**
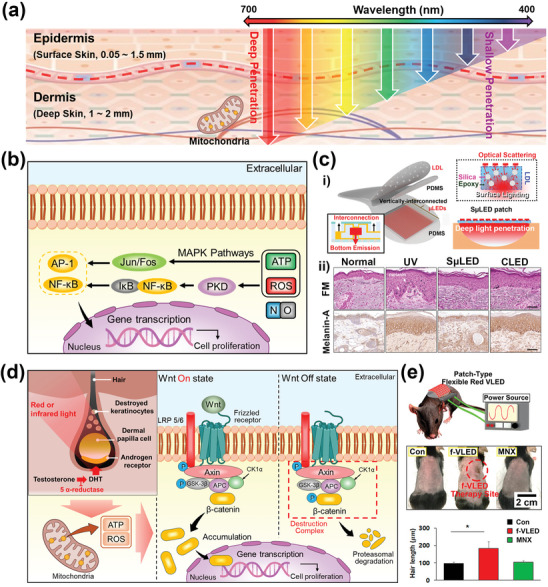
Cosmetic phototherapy via micro light‐emitting diodes (µLEDs). a) Schematic illustration of light penetration depth into the skin according to the wavelength of light. b) Conceptual illustration of the mechanism of skin anti‐aging through photobiomodulation (PBM) therapy. c) The surface‐lighting µLEDs (SµLED) patch for melanogenesis inhibition. i) The SµLED patch (left), radiation methods of the SµLED utilizing LDL (right), ii) the comparison of the result of in vivo experiment. Reproduced with permission.^[^ ^35^ ^]^ Copyright 2023, Wiley‐VCH. d) Conceptual illustration of the mechanism of alopecia treatment through PBM therapy. e) Photographs of mouse dorsal skin incontrol, µLED‐treated, and MNX‐treated groups after 20 days of hair‐regrowth experiments (upper). Comparison of hair growth length after treatments (lower) (^*^ *p* < 0.05, paired *t*‐test). Reproduced with permission.^[^ ^34^ ^]^ Copyright 2018, American Chemical Society. Some parts of Figure 3 were created with BioRender.com.

**Figure** **3**a illustrates the light penetration depth into the skin based on wavelength, showing that red light penetrates deeply and effectively stimulates the mitochondria in the dermis. The optical stimulation of mitochondria induces the activation of cytochrome *c* oxidase (CCO), leading to the production of adenosine triphosphate (ATP), reactive oxygen species (ROS), and nitric oxide (NO).^[^ ^28^, ^32^, ^33^ ^]^ The ATP synthesized through photostimulation activates cellular metabolism and energy, promoting collagen production and elastin synthesis through the metabolism of fibroblasts. The generation of low levels of ROS plays a role in maintaining redox homeostasis and can protect mitochondria from oxidative damage.^[^ ^105^ ^]^ As shown in Figure 3b, ROS triggers the mitogen‐activated protein kinases pathway that activates the AP‐1 transcription factor including c‐jun and c‐fos. Moreover, ROS plays a critical role in activating protein kinase D (PKD), leading to the degradation of IκB in NF‐κB/IκB complex, allowing NF‐κB transcriptional activation.^[^ ^106^, ^107^ ^]^ The activation of AP‐1 and NF‐κB enables skin anti‐aging by increasing the expression of genes related to collagen synthesis, anti‐inflammatory signaling, and cell migration and proliferation.^[^ ^108^ ^]^ Additionally, the released NO increases localized blood flow and enhances skin cell turnover, contributing to healthy and resilient skin.^[^ ^109^ ^]^

With the emergence of these mechanisms, several skincare photodevices have emerged utilizing red or near‐infrared light‐based lasers or LEDs. However, traditional devices with spot‐lighting face limitations such as light loss from the distance between the skin and the light source, nonuniform illumination, and lack of flexibility. Moreover, these noncontact type photonic devices with significant light loss face substantial challenges in achieving deep light penetration and high‐power efficiency even at high irradiance. To overcome these challenges, a flexible surface‐lighting µLEDs (SµLED) patch has emerged, providing conformal contact and uniform light irradiation with deep penetration depth through a silica‐based light diffusion layer (LDL). The uniformity of irradiance required for consistent therapeutic effects is influenced by several factors such as pixel spacing, distance and angle between the light source and the treatment site, and the scattering coefficient of tissue. Notably, since the curvature and structure of the skin vary among individuals, irradiance uniformity can change significantly depending on the distance and angle between the light source and the treatment site. To address this issue, the LDL, which is composed of silica and biocompatible polymers, conforms to the skin and induces optical scattering of the incident light, providing uniform light irradiation across all therapy sites (Figure 3c‐i). Moreover, due to the characteristics of conformal contact to the skin, the SµLED patch reduces light loss caused by light reflections at the LED‐skin interface, ensuring deeper light penetration depth and maximizing the effectiveness of PBM therapy.

To further investigate the effect of the SµLED patch on melanogenesis inhibition, a reliable in vivo animal experiment was conducted. An in vivo experiment process was divided into four experimental groups: i) a normal group (no treatment), ii) a group exposed only to UV, iii) a group exposed to both UV and SµLEDs, and iv) a group exposed to both UV and CLEDs. As shown in the FM‐stained images, the SµLED treatment group revealed a reduction in UV‐induced photodamage and confirmed the effective suppression of melanin production (Figure 3c‐ii).^[^ ^35^ ^]^ As a result, the mechanism for melanin production inhibition was confirmed, establishing the anti‐melanin effect of SµLEDs as an innovative solution for the skincare and cosmetics market.

#### 2.2.2 PBM Therapy for Hair Loss

Light‐based hair loss treatment has emerged to overcome the limitations of low safety in oral and topical treatments.^[^ ^110^, ^111^, ^112^ ^]^ As shown in Figure 3d, the mechanism of AGA treatment through PBM therapy using red or near‐infrared light begins with stimulating of CCO within the mitochondria located in dermal papilla cell, leading to the production of ATP, ROS, and NO. These result in the activation of the Wnt/β‐catenin pathway, which plays a crucial role in stimulating hair follicle morphogenesis and hair growth.^[^ ^34^, ^113^, ^114^ ^]^ The Wnt/β‐catenin signaling pathway is initiated when a protein called Wnt ligand binds to the Frizzled receptor and the LRP5/6 co‐receptor located on the cell surface.^[^ ^115^ ^]^ When the Wnt/β‐catenin signaling pathway is activated, β‐catenin, a protein that regulates critical signal transduction and gene expression within cells, accumulates without proteasomal degradation and moves to the nucleus. On the other hand, the Wnt/β‐catenin pathway is inactive, phosphorylated β‐catenin binds with Axin, GSK‐3β, APC, and CK1α to form the destruction complex, leading to degradation of β‐catenin.^[^ ^116^, ^117^ ^]^ The β‐catenin forms the complex with T‐cell factor/lymphoid enhancer factor, activating Wnt targeted gene expression.^[^ ^118^, ^119^ ^]^ This activates hair follicle stem cells (HFSCs), inducing the formation of new hair follicles and hair generation, and transitions dormant hair follicles into the anagen phase.

With the emergence of hair growth mechanisms utilizing light, µLEDs have been spotlighted as suitable light sources for hair loss treatment due to their excellent stability and flexibility. Figure 3e displays the successful hair growth in mice by PBM therapy utilizing µLED‐based patch‐type skin‐attachable photostimulator. To verify the efficacy of optical stimulator, the mice were treated for 20 days in three groups after shaving their hair on dorsal skin: a negative control group (maintained without any treatment), a positive control group (injection of minoxidil), and a µLED treatment group with periodic irradiation. The noticeable hair growth was achieved in the µLED treatment group, which exhibited the widest regrowth area. Moreover, the hair length of the mice treated with µLED was 183.2 µm, indicating a faster growth rate compared to 103.6 µm in the positive control group.^[^ ^34^ ^]^ This suggests that red light with a wavelength of 650 nm successfully stimulates hair growth by promoting the proliferation of HFSCs and entry into the anagen phase. Although the mechanisms for skin anti‐aging and hair loss treatment were confirmed by µLED‐based optoelectronics, the effects remain unproven across the entire facial and dorsal surface with complex curvature.

### 2.3 Medical Phototherapy

The demographics‐based predictions indicate that the number of new cases of cancer is expected to reach over 35 million by 2050.^[^ ^120^ ^]^ This increase is attributed to the growing population with major risk factors for cancer, such as smoking, being overweight, and obesity, which result from lifestyle changes in modern society. With the increasing incidence, cancer remains a significant challenge in clinical management. Treatment methods such as invasive surgery, chemotherapy, and radiation therapy have emerged for the management of cancer. However, the limitations including difficulty in accurate targeting and side effects hinder effective cancer therapy. PDT utilizing µLEDs has arisen as a promising alternative tumor treatment method that overcomes limitations and promotes the improvement of efficiency and safety.

The global incidence of neurological disorders such as stroke, Alzheimer's disease, and Parkinson's disease has been rising. These disorders can affect the nervous system throughout life by hindering brain development or damaging the brain, spinal cord, or peripheral nerves, leading to impairments in cognition, sensation, social‐emotional functioning, and motor functions and behavior.^[^ ^121^ ^]^ Therapeutic approaches including repetitive transcranial magnetic stimulation, and transcranial direct current stimulation have emerged for treating neurological disorders. However, these methods face limitations such as the requirement for repetitive treatments, resulting in longer therapy durations, and challenges in precise targeting, which hinder the achievement of consistent therapeutic effects.^[^ ^122^ ^]^ To overcome these limitations, optogenetic modulation through µLEDs has been reported, enabling immediate treatment effects and precise targeting.

#### 2.3.1 PDT for Cancer

**Figure 4:**
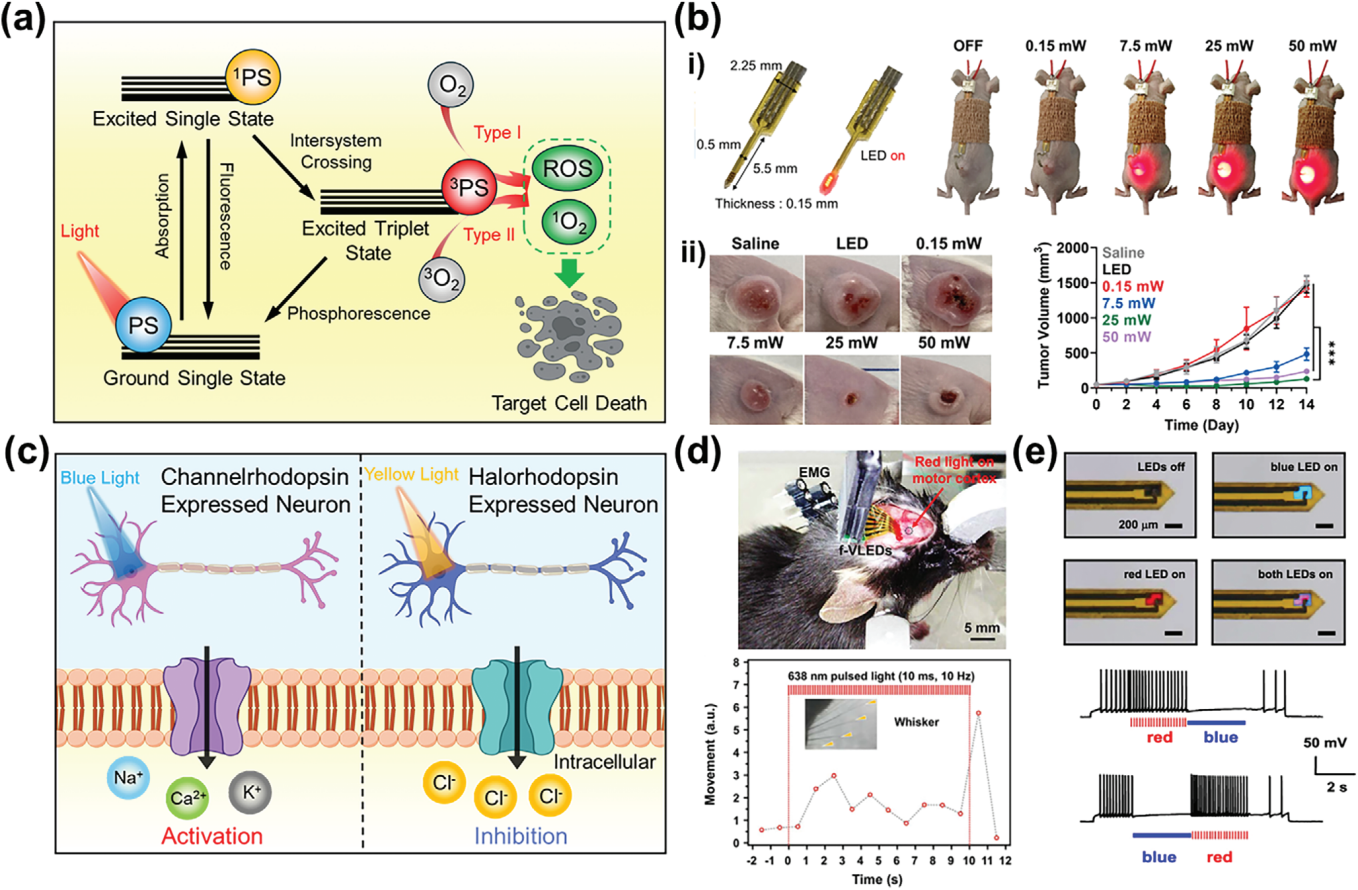
Medical phototherapy via micro light‐emitting diodes (µLEDs). a) Schematic illustration of the mechanism of photodynamic therapy (PDT) utilizing photosensitizer (PS). b‐i) Optical images and layout of the µLEDs (left), optical images of mice implanted with µLEDs for local visible light irradiation of tumor tissues (right), ii) Optical images of tumor volumes and collected tumor tissues of mice on day 14 after treatment (left), tumor growth of murine colon tumor models during µLED guided PDT at different light intensities (right). Reproduced with permission.^[^ ^43^ ^]^ Copyright 2022, Springer Nature. c) Schematic illustration of the mechanism of optogenetic modulation. d) Implantation of µLEDs beneath the skull to stimulate the motor cortex (upper). Enhanced muscle activities and body movements induced by µLED operation (638 nm, 10 Hz, 10 ms) (lower). Distance of whisker movement with µLED stimulation. Yellow triangles, the whiskers. Reproduced with permission.^[^ ^48^ ^]^ Copyright 2018, Elsevier. e) Optical images of a µLED probe (with a thickness of ≈120 µm and a width of ≈320 µm), showing independent control of blue and red emissions under current injections (upper). Traces presenting bidirectional spike activation and inhibition with alternating red and blue illuminations (Patching current +90 pA; Red: duration 3 s, 20 Hz, 10‐ms pulse, 2.2 mW mm^−2^; Blue: duration 3 s, 8.5 mW mm^−2^) (lower). Reproduced with permission.^[^ ^133^ ^]^ Copyright 2022, Springer Nature. Some parts of Figure 4 were created with BioRender.com.

The interaction between light and PS induces antimicrobial and minimally invasive treatment of cancers and certain nonmalignant lesions. PS is defined as a material capable of absorbing light with a specific wavelength, triggering photochemical or photophysical reactions.^[^ ^123^ ^]^ **Figure** **4**a presents two types of main mechanisms of the photodynamic reaction: Type I, and Type II. When a PS absorbs the light, it transitions to an excited triplet state, which is highly reactive. In a Type I reaction, the triplet state of the PS exchanges electrons or hydrogen with molecules within the cell to generate radicals, which react with oxygen to produce ROS, thereby increasing oxidative stress in the body. In a Type II reaction, singlet oxygen is generated through direct energy transfer between the PS and oxygen molecules in the triplet state.^[^ ^43^, ^124^ ^]^ Highly reactive ROS and singlet oxygen oxidize intracellular molecules, causing damage to cell membranes, mitochondria, and DNA, which ultimately induces either apoptosis or necrosis.

The precise match between the reactive wavelength of the PS and the emission wavelength of light sources is essential for highly efficacious PDT. PDT is divided into antimicrobial and cancer treatments depending on the wavelength of light, with blue light primarily used for surface antimicrobial PDT where deep penetration is unnecessary.^[^ ^125^, ^126^ ^]^ On the other hand, visible red and near‐infrared light, which penetrates deep tissue due to its longer wavelength, is mainly used for cancer treatment. However, cancers located deep within the body are not affected by direct irradiation from an external light source due to the limitations of light penetration depth. To overcome this challenge, as shown in Figure 4b, an implantable and biocompatible red µLED probe capable of direct light irradiation to deep‐seated tumors has been reported. Through the adjustment of the output power of µLEDs, the optimization of cell reaction was achievable (Figure 4b‐i). The irradiation of the red µLED with an intensity of over 25 mW induced necrotic cell death, whereas high early and late apoptosis of tumor cells was caused by the illumination of the light with an output power of 25 mW. The favorable patterns of cell death induced by the red µLED at optimized light intensity promoted a strong antitumor immunity in colon tumor‐bearing mice, resulting in a high rate of 40% complete tumor regression (Figure 4b‐ii).^[^ ^43^ ^]^ This treatment, which requires minimal invasiveness without surgical operation, can establish a position as a human‐centric and patient‐friendly cancer therapy.

#### 2.3.2 Optogenetic Modulation for Neurological Disorder Treatment

As shown in Figure 4c, light can induce activation or inhibition of neurons, mainly distributed in the brain and spinal cord through a photochemical reaction with light‐sensitive proteins known as opsins. Once opsin is expressed in the local cell area, it can absorb light, opening ion channels that allow ions to flow into the neuron, resulting in activation through depolarization and generation of an electrical signal.^[^ ^22^, ^48^, ^127^ ^]^ Conversely, opening ion channels that restrict ion flow into the neuron or closing ion channels can induce the inhibition of neuronal activity through hyperpolarization. The brain is composed of various functional regions such as the hippocampus, amygdala, and frontal lobe. Each region performs functions related to memory formation and spatial cognition,^[^ ^128^ ^]^ emotional processing,^[^ ^129^, ^130^ ^]^ decision‐making,^[^ ^131^ ^,^ ^132^ ^]^ and behavior.^[^ ^48^ ^]^ Notably, each region of the brain is distributed very finely, requiring precise targeting and allowing high‐resolution light illumination through the application of µLEDs.

Figure 4d shows the successful photostimulation of the motor cortex, responsible for body behavior, confirming light‐induced movement through a biocompatible neural implant with red µLEDs.^[^ ^48^ ^]^ After the expression of chrimson, red‐shifted channelrhodopsin, flexible µLED array was attached to the surface of the motor cortex in regions related to the jaw, wrist, neck, and whisker movement. During the irradiation of the pulsed red light with 10 Hz frequency, the electromyogram (EMG) signal and the behavior of whiskers and forelimbs were induced through photostimulation. Despite the device being implanted under the skull, the minimal tissue damage was confirmed through histological analysis. The behavioral response induced by optical stimulation of µLEDs paves the way for treating behavior‐related neurological disorders through optogenetic modulation.

Sequential activation and inhibition of neurons can be achieved through various wavelengths of light emitted from a single device. Figure 4e exhibits the bidirectional optogenetic modulation by a wireless dual‐color neural probe emitting light in three colors via a dielectric filter placed between the red and blue µLEDs.^[^ ^133^ ^]^ After the co‐expression of two distinct opsins (ChrimsonR and stGtACR2) in the rodent model, the probe was implanted into the ventral tegmental area. As a result, the bidirectional activation and inhibition of neurons in the same cell are demonstrated through momentarily altered red and blue light stimulation. Bidirectional neuronal control through the simultaneous expression of opsins enables more precise regulation of neuronal activity, providing an opportunity to explore complex brain circuits.

Beyond the brain, applications of optogenetic modulation are expanding to the entire body, including the spinal cord and organs.^[^ ^134^, ^135^, ^136^ ^]^ The spinal cord, which is linked to the brain and transmits sensory and motor nerve signals, plays crucial roles in controlling movement and basic physiological functions.^[^ ^137^, ^138^ ^]^ Optogenetic modulation through various organs such as the heart, stomach, and gut, regulates functions by stimulating neurons associated with the organs.^[^ ^136^, ^139^ ^]^ Optogenetic modulation is increasingly being used to study and influence these systems, offering new insights into body‐wide neural interactions and potential therapeutic applications.

## 3 Functionality and Strategies of 3D µLEDs for Human‐Centric Phototherapy

To realize human‐centric phototherapy, several requirements such as optical requirements, in vivo requirements, and structural design need to be fulfilled. The optical requirements such as wavelength, irradiance, and uniformity determine the light penetration depth in tissues, which are crucial factors influencing the efficacy of phototherapy. Moreover, in vivo requirements including heat generation and biocompatibility are critical for devices that establish direct contact with tissues. Notably, the consideration of wavelength, irradiance, and tissue‐specific absorption coefficient is essential to prevent tissue thermal damage during operation under conformal contact with tissue. To adapt organs and tissues with various shapes, sizes, and curvatures, the structural design of photonic devices plays a vital role in achieving conformal contact. The utilization of µLEDs in phototherapy offers superior optical properties, stability in biological environments, and flexibility, making them a promising solution for human‐centric phototherapy. Therefore, the development of µLED‐based photonic devices with structural design is essential for highly efficacious human‐centric phototherapy.

3D µLEDs refer to µLED devices with a three‐dimensional structure designed to adapt to complex 3D curved surfaces, respond to dynamic systems, and achieve functionalities unattainable with traditional 2D electronics. Their ability to overcome the inherent limitations of 2D systems makes them highly applicable in areas requiring advanced spatial adaptability, necessitating extensive research. While arrays of rigid or flexible devices can address certain constraints in phototherapy, fabricating and deploying multiple devices on intricate curved surfaces is often inefficient due to increased cost, time, and complexity. Although flexible 2D devices can be attached to organs and tissues, they are unable to achieve conformal contact across all surfaces of organs and tissues, resulting in a light loss that causes shallow light penetration and leads to diminished phototherapy efficacy. **Table** **5** shows the comparison of device characteristics including light penetration depth and treatment efficacy between 3D µLEDs and conventional 2D µLEDs.

**Table 5:** Comparison of the device characteristics between conventional two‐dimensional (2D) micro light‐emitting diodes (µLEDs) and three‐dimensional (3D) µLEDs.

Characteristics	Conventional 2D µLEDs	3D µLEDs
Contact capability	Nonconformal contact	Conformal contact
Form adaptability	Low	High
Spatial degree of freedom	Low	High
Light loss	High	Low
Light penetration depth	Shallow	Deep
Treatment efficacy	Low	High

Light‐delivery systems should be intentionally designed or programmed to direct light to target regions from diverse angles for achieving human‐centric phototherapy. This approach facilitates the realization of key phototherapeutic goals including uniform treatment of multiple spots or large areas in static environments, adaptive treatment in dynamic environments, and treatment of previously inaccessible spatiotemporal or multidimensional regions. Based on these objectives, the three representative functionalities of 3D µLEDs can be classified as 1) shape morphing, 2) self‐adaptation, and 3) spatiotemporal mapping. These functionalities facilitate highly efficacious and human‐centric phototherapy by providing deep light penetration, precise irradiation, and simultaneous multisite stimulation, respectively.

Applications of 3D µLEDs in human‐centric phototherapy, such as in neuroscience and cancer treatment, represent largely unexplored domains, posing challenges for classification under existing frameworks. This necessitates a functional categorization alongside strategies to achieve these functionalities, encompassing advancements in 3D structural design, material innovation, and enhancements in optical performance. This section addresses these aspects and provides perspectives on future research directions in this emerging field.

### 3.1 Shape Morphing

**Figure 5:**
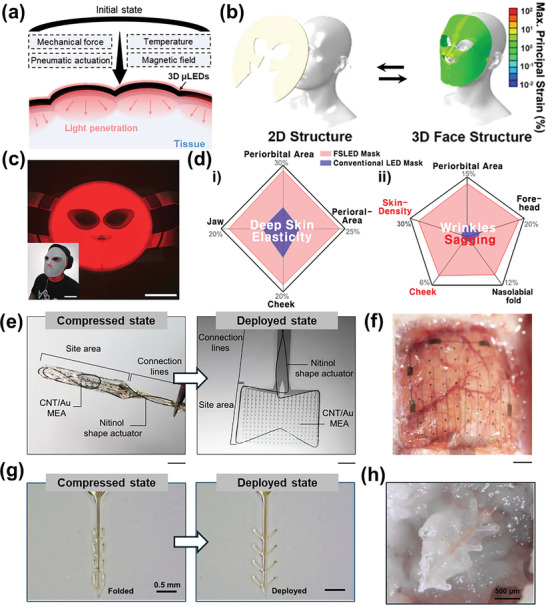
Shape morphing functionality and strategies of three‐dimensional (3D) micro light‐emitting diodes (µLEDs). a) Schematic illustration of the 3D µLED functionality of shape morphing. b) The two‐dimensional (2D) to 3D transformation procedure and strain distribution in the 3D face‐originated structure. c) Optical image of the face‐fit surface‐lighting µLED (FSLED) mask showing uniform surface‐lighting. The inset image is a photograph image of the FSLED mask fitted on a human face. Scale bar, 10 cm. d) Difference in cosmetic effect on deep skin elasticity between FSLED mask and conventional LED (CLED) mask. i) Deep skin elasticity, ii) Wrinkles and sagging. Reproduced with permission.^[^ ^33^ ^]^ Copyright 2024, Wiley‐VCH. e) Optical images of a shape‐changing electrode array (SCEA) in the compressed (left) and deployed states (right). Scale bar, 5 mm. f) A SCEA implanted epidurally into a rat brain through minimally invasive surgery. Scale bar, 1 mm. Reproduced with permission.^[^ ^149^ ^]^ Copyright 2024, Springer Nature. g) Optical images displaying the configurations of the probe in compressed state (left) and deployed state (right) at a temperature of 65 °C for 60 s. h) Optical image revealing the unfolded probe configuration of the deployable probe in the dissected rat brain. Reproduced with permission.^[^ ^150^ ^]^ Copyright 2024, National Academy of Sciences.

The human body comprises complex skin and organs with coexisting convex and concave curvatures, necessitating special strategies to utilize optoelectronic devices in biomedical therapeutic applications. Shape morphing is an effective strategy that enables the transformation of 2D electronic devices into 3D structures through various stimuli, such as mechanical force, pneumatics, temperature changes, light, or magnetic fields, allowing for enhanced conformal adhesion.^[^ ^140^, ^141^, ^142^ ^]^ This approach has been extensively researched in biomedical electronics, soft robotics, and deployable systems. As shown in **Figure** **5**a, 3D µLEDs designed for human‐centric phototherapy achieve conformal adhesion on tissue surfaces of all shapes, minimize stress on biological tissues, and provide uniform treatment over deep and broad areas. 3D µLEDs with shape morphing capabilities can be classified into wearable and implantable forms, with ongoing research focusing on design optimization and 2D‐to‐3D transformation mechanisms to implement shape morphing functionality.^[^ ^143^ ^]^

For wearable forms of 3D µLEDs, applicable in areas including skincare and wound healing, the primary objectives achieved through shape morphing are providing a user‐friendly wearing experience and delivering high‐efficiency deep light penetration via conformal adhesion without tissue light attenuation.^[^ ^35^, ^144^ ^]^ A notable example is a face‐fit surface‐lighting µLED (FSLED) mask designed for skin rejuvenation. As presented in Figure 5b, the FSLED mask incorporates a face‐shaped origami design to conform to the complex 3D geometry of the human face while maintaining a consistent 3% strain across its entire surface. As depicted in Figure 5c, with an array of 3770 red µLEDs and LDL, the mask delivers uniform light treatment capable of penetrating up to 1.5 mm into the dermis, ensuring effective and consistent therapeutic outcomes. Clinical trial demonstrated significant improvements in elasticity, sagging, and wrinkles across six facial areas compared to the conventional LED mask group. The improvement in deep skin elasticity, which requires light to penetrate deeply into the dermis, showed an impressive increase of up to 340% (Figure 5d‐i). Wrinkle and sagging improvements reached up to 270%, and pore size and skin density improvements up to 230% (Figure 5d‐ii).^[^ ^33^ ^]^ These remarkable improvements are attributed to the ability of the FSLED mask to deliver light more effectively to the skin, promoting elastin and collagen synthesis and cell regeneration.

For implantable 3D µLEDs, preventing device degradation during the implant procedure is essential. To address this issue, diverse strategies including serpentine‐shaped electrodes and self‐healing materials have recently been developed.^[^ ^145^, ^146^ ^]^ Moreover, key objectives of implantable 3D µLEDs include minimizing tissue damage by reducing invasiveness and achieving efficient light delivery to targeted regions across large areas and multiple angles. Advanced material designs that enable controlled shape morphing offer a promising approach to achieve the necessary 2D‐to‐3D transformations for implementing 3D µLED systems. Recent developments have focused on a variety of actuation materials, such as shape memory alloys (SMAs), shape memory polymers (SMPs), hydrogel swelling mechanisms, and pneumatic systems.^[^ ^147^, ^148^, ^149^, ^150^ ^]^ Among these, SMAs and SMPs are particularly notable for their temperature‐responsive shape memory effects, allowing deformation and recovery triggered by body temperature without external assistance. For example, a flexible shape‐changing electrode array (SCEA) utilizing nitinol, a widely used SMA, has demonstrated the potential of 3D µLEDs as large‐area optogenetic implantable devices. Nitinol, a nickel‐titanium alloys, undergoes a phase transformation from martensite (low‐temperature stable phase) to austenite (high‐temperature stable phase) when heated above its transition temperature, enabling it to recover its pre‐programmed shape and size. The SCEA, composed of 64 carbon nanotube/gold mesh electrode arrays, was compressed from 20 × 15 mm to a 3 mm‐wide strip using the water‐soluble adhesive polyethylene oxide. Figure 5e exhibits the SCEA that reverts to its original shape upon reaching the phase transition temperature of 37 °C, enabling seamless expansion. As depicted in Figure 5f, the shape morphing feature of the SCEA facilitates insertion through a small opening in the skull or dura mater, where the device expands to cover a large cortical area.^[^ ^149^ ^]^

Another compelling example involves a deployable intracortical probe leveraging a thiol‐ene/acrylate‐based SMPs, highlighting the potential of 3D µLEDs as high‐resolution neuromodulation probes. SMPs demonstrate excellent biocompatibility and unique thermal properties, transitioning to a rubbery state with a modulus of 10 MPa when heated to its glass transition temperature of 58 °C. At ambient temperature, the probe maintains a significantly higher modulus of 2.3 GPa, allowing it to be mechanically folded into a compact configuration for minimally invasive implantation. After implantation, body temperature triggers the SMPs to restore its pre‐programmed shape, expanding the branching electrodes to interface with a wider neural region. Figure 5g exhibits the fishbone‐like structure with branching electrodes where the electrodes expand via the SMP to record signals over a wider area. As shown in Figure 5h, this design reduces mechanical mismatch with brain tissue, achieving long‐term stable implantation and recording neural signals at single‐neuron resolution.^[^ ^150^ ^]^ Applying this strategy to 3D µLEDs promises to enhance the efficacy of optogenetic therapies by enabling precise stimulation of targeted neural circuits.

### 3.2 Self‐Adaptation

**Figure 6:**
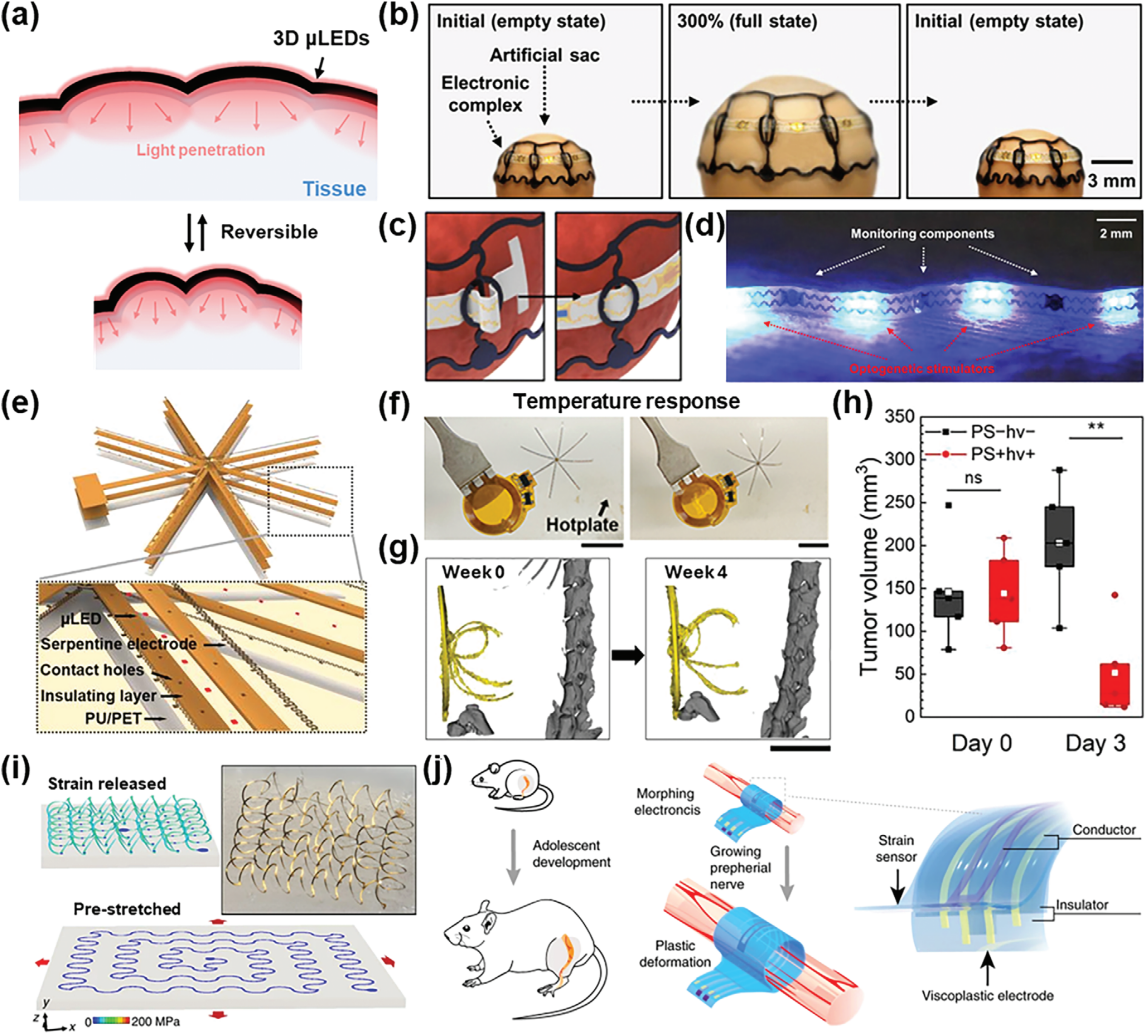
Self‐adaptation functionality and strategies of three‐dimensional (3D) micro light‐emitting diodes (µLEDs). a) Schematic illustration of the 3D µLED functionality of self‐adaptation on tissue with reversible volume changing. b) Images of artificial distensible sacs wrapped with the expandable electronics, emulating the repetitive expansion (300% in volume) and contraction motions of the bladder. c) The schematic of E‐thread in construction state (left) and expanded state (right). d) An optical image of the E‐thread, built with eight µ‐LEDs distributed onto four islands with three monitoring components. Reproduced with permission.^[^ ^154^ ^]^ Copyright 2020, AAAS. e) A schematic image of shape‐morphing µLED (SMLED) structure. f) The shape changes of SMLED in response to temperature, scale bar 1 cm. g) Reconstructed CT images of a nude mouse taken over a period of 4 weeks following the implantation of mPDT system, scale bar 1 cm. h) Measured tumor volumes at Day 0 and Day 3 post‐treatment. Box plots show the interquartile range with whiskers representing outliers up to 1.5 times the interquartile range. Mean values are indicated by square symbols within each box, and medians are represented by solid horizontal lines (*n* = 5 per condition). Statistical significance between groups on Day 3 is indicated as ^**^ *p* < 0.01. Reproduced with permission.^[^ ^52^ ^]^ Copyright 2024, Wiley‐VCH. i) Process for assembly illustrated by finite element analysis (FEA). A two‐dimensional (2D) filamentary serpentine structure bonded at selected locations to an underling, bi‐axially stretched (*ɛ*_pre_) soft elastomeric substrate (pre‐streched). Corresponding 3D helical coils formed by relaxation of the substrate to its initial, unstretched state (strain released). The color represents the magnitude of Mises stress in the metal layer. Inset: Angled optical images of an experimentally realized structure. Reproduced with permission.^[^ ^155^ ^]^ Copyright 2017, Springer Nature. j) Schematics showing adolescent development of the rat and the multilayered morphing electronics (MorphE) that conformally adapts to sciatic nerve growth. MorphE allows plastic deformation with little stress when stretched by a growing nerve. Reproduced with permission.^[^ ^156^ ^]^ Copyright 2020, Springer Nature.

Real‐time adaptation to dynamic tissues, such as the heart, bladder, and tumors, is a critical requirement for optoelectronic devices. These properties focus on facilitating continuous deformation to provide more accurate light delivery in different environment changes. The conventional adapting of devices with tissues relies on invasive insertion, surgical suturing, and physical adhesion using chemical adhesive to ensure attachment. However, these approaches face challenges such as nonconformal contact, unstable adhesion, and tissue damage, caused by deformations, including tissue volume expansion and contraction.^[^ ^151^ ^]^ An effective way to enhance device adaptability and conformability without additive methods is to design self‐adaptive 3D devices.^[^ ^52^, ^152^ ^]^ **Figure** **6**a shows the self‐adaptive property which is the ability to reversibly reconstruct 3D µLED shape according to the tissue deformation, ensuring continuous conformal light delivery. Self‐adaptive 3D µLEDs that can be continuously actuated via external environment changes, such as temperature, humidity, and volume change, hold great importance for future applications in implantable and wearable devices.^[^ ^153^ ^]^

A variety of strategies have been investigated to improve the ability of 3D µLEDs to adapt to environmental changes. Notable studies include the development of expandable bioelectronics complex and shape‐morphing µLED (SMLED). Figure 6b displays the self‐adaptive function of an expandable bioelectronic complex for analyzing and regulating real‐time activity of the urinary bladder. An expandable bioelectronic complex, consisting of web‐type stretchable framework and thread, not only envelops the urinary bladder across entire surface but also maintains intimate contact with the distensible sac during substantial volume changes. This capability is derived from novel design of electronic complex. Figure 6c exhibits two structural components that facilitate smooth expansion and contraction by allowing the threads connected to the E‐web to rearrange as the bladder stretches and contracts over the course of several minutes. Based on stable self‐adaptive functionality, this expandable system demonstrates stable operation of LED and monitoring components even after the rapid volumetric changes in the bladder, as shown in Figure 6d.^[^ ^154^ ^]^

Similarly, Figure 6e displays the SMLED for pancreatic cancer treatment. This device utilizes a 5‐micrometer‐thick µLED to implement a thin, 15‐micrometer device, which can easily morph its shape. Additionally, as depicted in Figure 6f, the aspect ratio is increased by over 15 times to maximize thermal deformation caused by external temperature changes. As illustrated in Figure 6g, the 3D µLEDs demonstrated self‐adaptive capabilities during continuous volume expansion and contraction throughout the treatment process, effectively delivering light to the surface of the pancreatic tumor over a four‐week treatment period. Due to this self‐adaptive capability, SMLEDs enable real‐time adaption to aggressively growing cancers, providing precise light irradiation to tumors and allowing for accurate cancer treatment without damaging surrounding normal tissues. As shown in Figure 6h, the mPDT therapy utilizing the self‐adaptive SMLED achieved a significant reduction in pancreatic ductal adenocarcinoma (PDAC) volume from 144.3 ± 51.9 to 51.6 ± 54.5 mm^3^, highlighting a 64% reduction, while avoiding harmful effects on surrounding tissues.^[^ ^52^ ^]^ This self‐adaptive functionality, induced by µLEDs and a 3D high aspect‐ratio origami design, represents a promising advancement in three‐dimensional phototherapy, offering a safe and targeted approach.

To further advance 3D self‐adaptive functionality, improvement strategies are required not only in design modifications but also in processes and materials. One of the key approaches in self‐adaptive technique is the development of 3D electrodes through novel fabrication processes. Figure 6i displays the 3D helical electrode that provides a uniform distribution of deformation‐induced stresses, which reduces the risk of local cracks or fractures and enables high stretchability and mechanical robustness. The fabrication of this 3D electrode involves bonding the ends of a 2D precursor to an elastomeric silicone substrate using strong covalent siloxane bonds under biaxial pre‐strain, while other regions adhere through weak van der Waals interactions. Upon releasing the pre‐strain, compressive forces induce controlled deformations, transforming the precursor into a 3D helical electrode through coordinated motions.^[^ ^155^ ^]^ In continuously deforming 3D self‐adaptive systems, the incorporation of 3D electrodes with µLEDs enhances the endurance of the devices, allowing them to adapt to various shape‐morphing with deformations. Another key approach for advancement is the development of material design. Figure 6j illustrates multilayered morphing electronics (MorphE) with growth‐adaptive properties. MorphE combines viscoplastic material design tailored to tissue growth rates with self‐healing technology to create electronic devices capable of adapting to tissue growth.^[^ ^156^ ^]^ This approach minimizes mechanical stress, ensures long‐term stability and biocompatibility, and demonstrates the potential for full‐cycle phototherapy using self‐adaptive characteristics.

### 3.3 Multilayered Spatiotemporal Mapping

**Figure 7:**
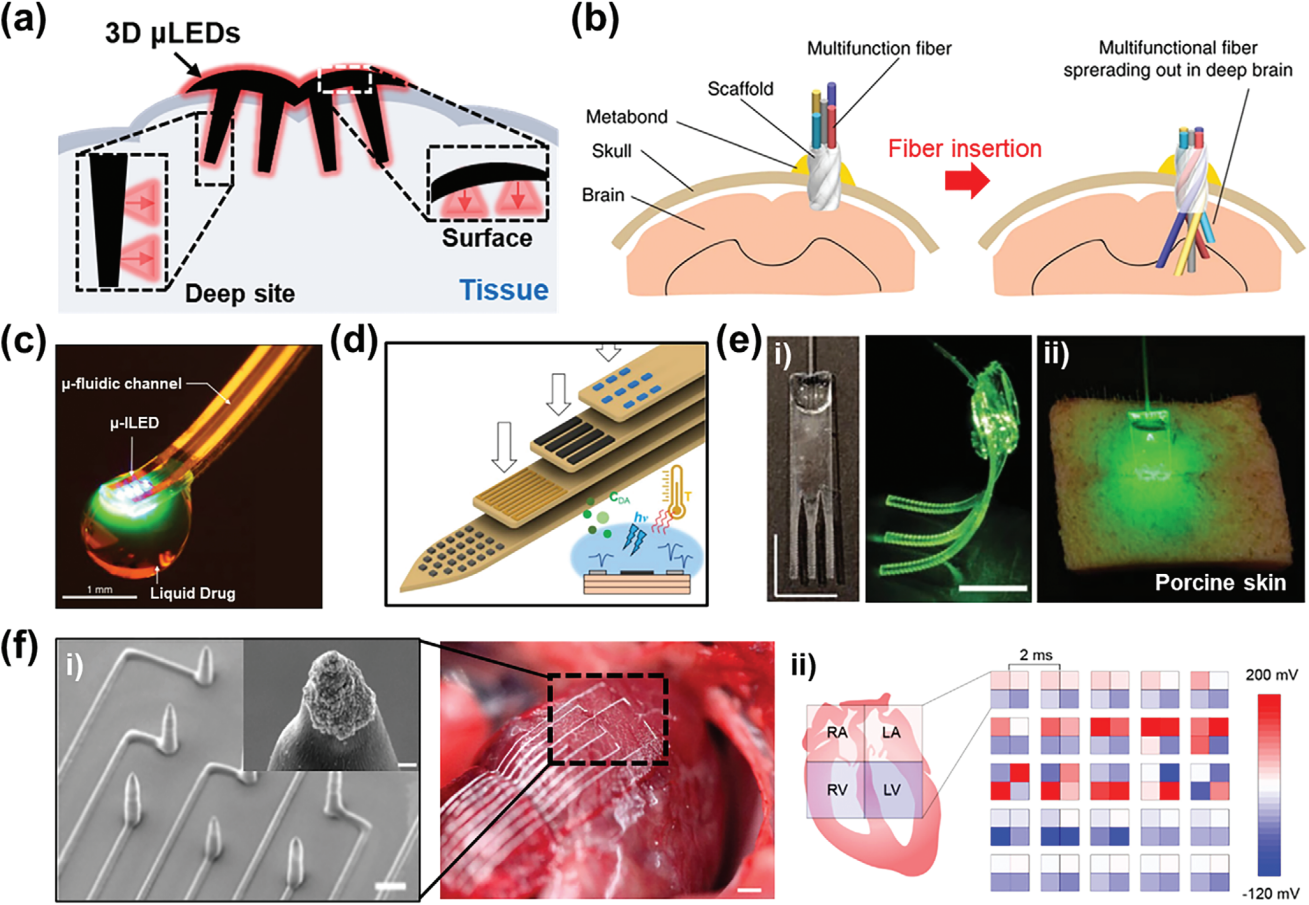
Multilayered spatiotemporal mapping functionality and strategies of three‐dimensional (3D) micro light‐emitting diodes (µLEDs). a) Schematic illustration of the 3D µLED functionality of multilayered spatiotemporal mapping. b) Scaffolding fiber is inserted into the brain (left), functional fiber probes are further inserted into the brain tissue through the scaffolding fiber (right). Reproduced with permission.^[^ ^159^ ^]^ Copyright 2020, Springer Nature. c) Optofluidic neural probe during simultaneous drug delivery and photostimulation. Reproduced with permission.^[^ ^160^ ^]^ Copyright 2015, Cell Press. d) Schematic illustration of multifunctional neural probe composed of recording site, dopamine sensor, and a temperature sensor. Reproduced with permission.^[^ ^161^ ^]^ e‐i) Waveguide bundle engineered for optimal light delivery to the tissue (left), waveguide array carrying green laser light. Scale bars, 10 mm (right). ii) Waveguide inserted in porcine skin incision illuminated through a fiber by a green laser. Reproduced with permission.^[^ ^162^ ^]^ Copyright 2016, Springer Nature. f‐i) SEM image of the 3D liquid electrode array. Scale bar, 500 µm (magnified image), Photograph of 3D liquid electrodes transferred to the rabbit heart, 5 mm (right). ii) Heart electrical mapping data measured at 2 ms intervals from each atrium and ventricle. Reproduced with permission.^[^ ^163^ ^]^ Copyright 2024, American Chemical Society.

Multilayered spatiotemporal mapping is a powerful functionality of 3D µLEDs, enabling the analysis of changes in complex biological systems such as neural networks, physiological responses, and cellular interactions within a 3D space. However, conventional probe‐based or flat 2D devices face spatial limitations, allowing spatiotemporal mapping only within restricted areas through either deep insertion or surface attachment.^[^ ^48^, ^68^, ^157^ ^]^ Although the noninvasive optical brain stimulation method with 2D optoelectronics was reported, this method faces significant limitations such as inaccurate stimulation of multiple deep brain regions, requirement of high light intensity due to light scattering, and concerns about tissue damage.^[^ ^158^ ^]^ The 3D µLEDs, which introduce an insertion component formed in the z‐axis direction on a flat device, enable simultaneous attachment and implantation into tissues. Despite the invasiveness for muti‐site and deep‐site optical stimulation, 3D µLEDs allow for precise optical stimulation without high light intensity and critical damage, overcoming the spatial limitations of conventional 2D optoelectronics. Multilayered spatiotemporal mapping paves the way for exploring unknown areas within human tissue and offers a superior approach to precisely elucidating the systems underlying complex brain circuits. Notably, high‐resolution multilayered spatiotemporal mapping through optogenetic modulation utilizing small‐sized µLEDs facilitates a clear analysis of interactions between single neurons, allowing for detecting early signs of neurological disorder or precise tracking of disease progression. **Figure** **7**a displays the schematic of the multilayered spatiotemporal mapping functionality of 3D µLEDs with concurrent photostimulation at the surface and deep site of tissue.

Diverse approaches have been investigated to enhance the capability of 3D µLEDs in multilayered spatiotemporal mapping. Figure 7b exhibits the schematic of spatially expandable multifunctional fiber probes. These probes were precisely inserted into deep brain regions at specified angles, enabling simultaneous photostimulation, electrical signal recording, and drug delivery across multiple brain areas. Integrating diverse functions into the insertion part of 3D µLEDs enables minimally invasive one‐step surgery, reducing treatment time and minimizing side effects. Notably, the simultaneous optical stimulation and electrical signal recording allow real‐time monitoring of tissue responses, enabling immediate feedback and facilitating precise optogenetic modulation.^[^ ^159^ ^]^ As shown in Figure 7c, simultaneous optical stimulation and fluid injection utilizing the soft optofluidic probe allow spatiotemporal control of both drugs and light, holding potential for application in multilayered spatiotemporal mapping.^[^ ^160^ ^]^ Figure 7d shows a multifunctional flexible probe integrated with a high‐density recording area, dopamine sensors, and temperature sensors. This probe allows simultaneous high spatial‐resolution electrophysiological signal recording, local temperature sensing, and dopamine concentration monitoring.^[^ ^161^ ^]^ By incorporating these functionalities, the capability of 3D µLEDs for multilayered spatiotemporal mapping can be enhanced, enabling disease diagnosis and treatment as well as real‐time monitoring.

Notably, 3D µLEDs require a low modulus to minimize cellular damage because they establish direct contact with soft biological tissues during insertion. Figure 7e shows the flexible and bioresorbable polymer‐based optical waveguide for deep tissue phototherapy. For the deep site photostimulation, the insertion part of the optical waveguide was formed by high‐precision laser cutting. The green laser light emission was observed along the entire region of the waveguide, whereas the laser light penetrates less than 5 mm without the waveguide (Figure 7e‐i). To further investigate its light delivery capability during the implant procedure, the waveguide was inserted in porcine skin, showing effective light coupling throughout the entire area (Figure 7e‐ii).^[^ ^162^ ^]^ This suggests that overcoming the limitations of light penetration depth in conventional light sources enables efficient light delivery, facilitating its application in diverse phototherapy fields with spatiotemporal mapping functionality.

The 3D liquid metal electrodes with low mechanical modulus and low freezing point enable precise and stable implantation into biological tissues for multilayered spatiotemporal mapping. Figure 7f presents a 3D liquid electrode array for spatiotemporal cardiac mapping and modulation. The 3D liquid electrode EGaIn electrode array was formed vertically by applying pneumatic pressure to the metal nozzle, with a Pt nanocluster to enhance the signal recording performance. Due to high biocompatibility and modulus matching with soft tissues, the 3D liquid electrode array was successfully implanted into the heart (Figure 7f‐i). To investigate the capability of electrical mapping using a 3D liquid electrode array, individual ECG signals at the atria and ventricles were recorded (Figure 7f‐ii).^[^ ^163^ ^]^ The liquid electrode array with the submillimeter scale enables precise targeting of the small atria and ventricles, paving the way for high‐resolution spatiotemporal mapping of 3D µLEDs.

### 3.4 Perspectives on Practical Challenges of 3D µLEDs for Human‐Centric Phototherapy

The 3D architecture with µLEDs for efficacious phototherapy can be realized through diverse designs with the potential to be applied to numerous body systems such as the head, brain, stomach, and intestine. Notably, the matrix of 3D µLEDs should be soft and flexible to facilitate 3D deformation, allowing conformal contact even with complex 3D curvatures. Moreover, it should be ensured high biocompatibility in terms of materials and structure to effectively adapt well to the biological environment and operate stably over an extended period without performance degradation. However, several challenges still remain in applying 3D µLEDs to humans.

Devices or methods used in preclinical tests including animal experiments and in vitro tests face diverse obstacles during their application to humans. These challenges arise from the differences in the size, shape, and curvature of organs and tissues between humans and animals, as well as complex biological variables in humans such as skin structure, metabolic systems, and inflammatory responses.^[^ ^164^, ^165^ ^]^ To address these issues, for example, optimizing the optical properties of 3D µLEDs according to the changes in skin structure or tumor size and density is essential in PBM therapy and PDT, respectively. Moreover, the structure of 3D µLEDs needs to be optimized for precise targeting of the more complex brain structure and for minimizing tissue damage during device implantation to enable the application of optogenetic modulation to humans.^[^ ^166^ ^]^ Beyond these therapeutic requirements of 3D µLEDs, substantial practical challenges remain to be addressed for human‐centric phototherapy.

**Figure 8:**
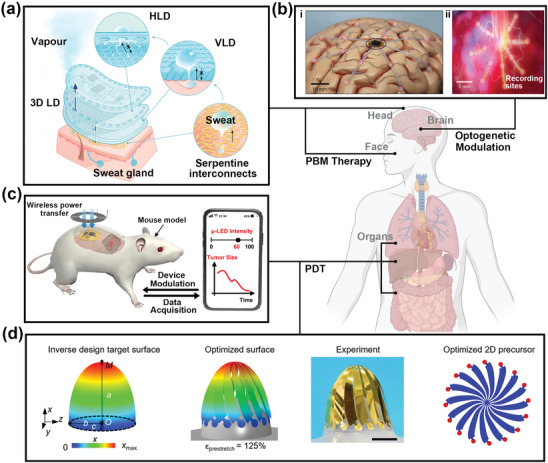
Perspectives on strategies for human‐centric phototherapy via three‐dimensional (3D) micro light‐emitting diodes (µLEDs). a) Schematic of the integrated system‐level sweat‐permeable electronics, consisting of permeable electrodes, 3D LD. Exploded view illustrates the unidirectional sweat transport through the electrode, vertical LD (VLD), and horizontal LD (HLD). Reproduced with permission.^[^ ^167^ ^]^ Copyright 2024, Springer Nature. b) Photograph of the biodegradable electronic tent (left), images of the deployed electronic tent during injection under a transparent skull replica (right). Reproduced with permission.^[^ ^168^ ^]^ Copyright 2024, Springer Nature. c) Schematic illustration of cancer treatment and monitoring system including device and programmable µLED intensity control and real‐time monitoring of tumor size, and chemical mechanism of PDT. Reproduced with permission.^[^ ^169^ ^]^ Copyright 2023, Springer Nature. d) Inverse design process for a hemi‐ellipsoidal surface assembled on the hemispherical substrate. Reproduced with permission.^[^ ^170^ ^]^ Copyright 2022, AAAS. Some parts of Figure 8 were created with BioRender.com.

The home‐care capable 3D µLEDs for hair loss treatment or skin aging improvement need to be designed to address heat generation, sweat secretion, and byproducts that occur during daily activities. **Figure** **8**a shows the concept of permeable electronics utilizing 3D liquid diodes (LDs) which enable unidirectionally self‐pumping sweat from the skin. By forming a hydrophilicity gradient channel in vertical LD (VLD), sweat generated at the skin is pumped out rapidly. To discharge sweat externally, horizontal LD (HLD) with gradient hydrophilic micropillars, where the transport speed increases as the spacing decreases, provide the capability to move sweat unidirectionally.^[^ ^167^ ^]^ The emergence of LDs integrating VLD and HLD proposed a new paradigm for permeable electronics, providing the potentials for the development of permeable 3D µLEDs.

For optogenetic modulation in the human brain, which is significantly larger than the brains of animals including mice or monkeys, optical stimulation across broad regions and minimally invasive procedures are required. Figure 8b exhibits a self‐deployable electronic tent electrode for brain cortex interfacing. This electronic tent achieves conformal contact with the brain surface, performing electrical functions such as temperature, pH, and strain sensing. When the device is inserted through the minimally pierced skull to the surface of the brain, the electronic tent automatically spreads in the radial direction by temperature‐dependent shape memory force.^[^ ^168^ ^]^ By applying this mechanism to 3D µLEDs, wide‐area optogenetic modulation and minimally invasive surgery become simultaneously feasible. Moreover, these 3D µLEDs enable the identification of interactions, correlations, and the brain circuitry system among various regions of the human brain including the temporal and occipital lobes.

The monitoring techniques for tumor volume changes during long‐term PDT are crucial for evaluating the accuracy of photodynamic diagnosis and the efficacy of PDT for human cancer treatment. Inaccurate detection of tumor volume can lead to recurrence after treatment or thermal damage to the tissue. Figure 8c displays the illustration of wireless real‐time monitoring and device modulation systems during PDT. The Si phototransistor located near the µLED can measure the emitter current according to the amount of light scattered through the tumor to detect changes in tumor size. Moreover, the adjustment of µLEDs intensity can be achieved by the user, depending on the progression of tumor treatment, using a custom‐designed Android application.^[^ ^169^ ^]^ Wireless real‐time monitoring of the treated tumor size, and intensity control of µLEDs can provide the potential to achieve continuous and patient‐friendly PDT.

The precise design of customized 3D curvatures of human tumors on a planar substrate poses significant challenges due to complex geometric shapes and uneven strain distribution. To overcome this issue, inverse designs of 3D surfaces with topology optimization based on curvature effects have emerged. Figure 8d exhibits the inverse design process and results for the hemi‐ellipsoidal surface assembled on a hemispherical substrate. It compares the target hemi‐ellipsoidal surface assembled on a hemispherical substrate with the experimentally optimized surface, showing that the assembled 3D surface structure aligns with the design target by utilizing predefined loads and pre‐stretched elastic substrates.^[^ ^170^ ^]^ The 3D µLEDs achieved through this inverse design provide effective treatment and stable adhesion regardless of the curvature and surface shape of anatomical areas, paving the way for expanded applicability in the medical and healthcare fields.

## 4 Conclusion

While significant progress has been made in phototherapy technologies, including PBM therapy, PDT, and optogenetic modulation, a comprehensive conceptual framework for achieving effective and human‐centeric phototherapy remains undeveloped. This perspective article highlights the potential of 3D µLEDs to advance human‐centric phototherapy by enabling deep light penetration, precise irradiation, and simultaneous multisite stimulation. In particular, recent research trends in shape morphing, self‐adaptation, and multilayered spatiotemporal mapping of 3D µLEDs are reviewed, in addition to proposed strategies and insights to enhance these functionalities. Subsequently, the practical challenges and solutions for the human‐centric phototherapy applications of 3D µLEDs are discussed, emphasizing the need for permeable, self‐deployable structures, wireless real‐time monitoring systems, and an approach from the perspective of inverse 3D design processes. In conclusion, 3D µLEDs, with superior light delivery and biocompatibility, hold great potential for cosmetic care, refractory disease treatment, and neuroscience, and ongoing research will guide next‐generation, patient‐friendly optoelectronic phototherapy.

## Conflict of Interest

The authors declare no conflict of interest.

## Author Contributions

K.Y.N., M.S.K., and J.A. contributed equally to this work. K.Y.N., M.S.K., J.A., and K.J.L. conceptualized the manuscript. K.Y.N., M.S.K., J.A., S.M., J.H.L., and J.S.P. reviewed the relevant literature and wrote the manuscript. C.‐H.H., S.H.Y., and K.J.L. edited and finalized the manuscript.
